# Inclusion of Sainfoin in the Concentrate of Finishing Lambs: Fatty Acid Profiles of Rumen, Plasma, and Muscle

**DOI:** 10.1021/acs.jafc.3c05902

**Published:** 2023-11-13

**Authors:** Clàudia Baila, Margalida Joy, Juan Ramón Bertolín, Susana Alves, Rui Bessa, Mireia Blanco, Sandra Lobón

**Affiliations:** † Departamento de Ciencia Animal, Centro de Investigación y Tecnología Agroalimentaria de Aragón (CITA), Avda. Montañana 930, Zaragoza 50059, Spain; ‡ Instituto Agroalimentario de Aragón − IA2 (CITA-Universidad de Zaragoza), Zaragoza 50059, Spain; § CIISA, Centro de Investigação Interdisciplinar em Sanidade Animal, Faculdade de Medicina Veterinária, Universidade de Lisboa, Lisboa 1300-477, Portugal; ∥ Laboratório Associado para Ciência Animal e Veterinária (AL4AnimalS), Avenida da Universidade Técnica, Lisboa 1300-477, Portugal

**Keywords:** lipid metabolism, local forage legume, meat quality, *Onobrychis viciifolia*, rumen biohydrogenation

## Abstract

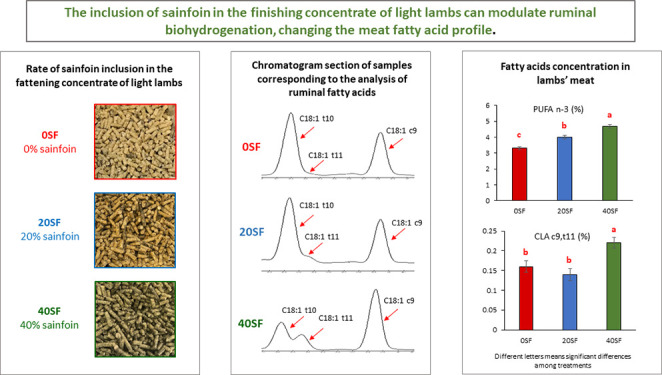

The effects of sainfoin inclusion (*Onobrychis viciifolia*) in the finishing concentrate for light lambs on the fatty acid (FA) composition of the ruminal digesta, plasma, and meat were evaluated. Twenty-six weaned male lambs were divided into three groups and fed individually *ad libitum* for 40 days with one of three concentrates differing in the level of sainfoin inclusion: 0% (0SF), 20% (20SF), and 40% (40SF). The rumen digesta showed an increase in C18:3 n-3 concentration and a decrease in C18:1 t10 concentration when sainfoin was included in the concentrate regardless of the level of inclusion. However, the highest C18:1 t11 and the lowest C18:2 n-6 proportions were obtained only in the 40SF rumen, showing a stronger t11 biohydrogenation pathway. In plasma, most effects were associated with changes in the levels of polyunsaturated FA (PUFA) n-3. The meat FA profile of 40SF lambs presented higher percentages of PUFA n-3 and CLA c9,t11 and a lower PUFA n-6/PUFA n-3 ratio compared with those from 0SF and 20SF diets because of the potentiation of the ruminal t11 pathway. Inclusions of 20 and 40% sainfoin both showed beneficial effects on meat quality; furthermore, these effects were most marked in the 40% sainfoin diet.

## Introduction

Current guidelines advise monitoring the intake of ruminant meat because of the high concentrations of saturated fatty acids (SFA),^1^ although this has a healthier n-6/n-3 FA ratio than that of pork or chicken.^2^ The high concentration of SFA in ruminant edible products is related to the ruminal biohydrogenation (BH), a complex process comprising multiple pathways that sequentially isomerize and hydrogenate the dietary unsaturated FAs, producing a more SFA profile.^3,4^ Although this might appear to compromise the current consumer desire to have healthier animal products in their diets,^5^ this microbial process produces beneficial bioactive FAs such as C18:1 t11 and C18:2 c9,t11 that are mostly related to ruminant products consumption.^6,7^ Hence, a deep understanding of the ruminal BH is needed to know and anticipate how thus can affect the metabolic availability of FA and their deposition in tissues and milk.^8^

Light lamb production systems in several Mediterranean countries, such as Spain and Portugal, are based on indoor concentrate-fed from weaning (12 to 14 kg of BW) to slaughter (22 to 28 kg of BW and 70–90 days old). The inclusion of locally produced forages has been commonly studied as a strategy to simultaneously achieve greater sustainability and self-sufficiency in intensive systems and improve animal welfare and the meat FA profile.^9,10^ Forages are rich in C18:3 n-3 and promote the BH pathway to produce C18:1 t11 instead of the formation of C18:1 t10 isomer, which is associated with concentrate-rich diets,^11^ producing more beneficial meat for consumers.

Sainfoin (*Onobrychis viciifolia*) is a Mediterranean forage legume with a high crude protein concentration and with a medium content of proanthocyanidins (PACs),^12^ also known as condensed tannins. Sainfoin is commonly preserved because its production is mainly obtained in the first spring cut. Thus, in this type of production system, it would be interesting to preserve sainfoin as pellets for inclusion in concentrates. In addition to the changes in ruminal BH caused by the inclusion of a forage in the concentrate, the presence of PACs can have a modulating effect on the specific BH pathways or the rumen microbial population. This can produce changes in the concentration of polyunsaturated FAs (PUFAs) and FA intermediates (C18:3, C18:2, and C18:1 isomers) in the rumen,^13^ which are potentially deposited on to ruminant products, such as meat.^14^

We hypothesized that including dehydrated sainfoin forage into finishing diets for light lambs would promote the rumen BH pathways producing C18:1 t11 and C18:2 c9,t11 and thereby improve the FA profile of lamb meat. The study sought to evaluate the effects of the inclusion of sainfoin at two different rates in the finishing pelleted concentrate for lambs on the ruminal BH and on blood and meat FA profiles. Results from this experiment will provide a better understanding of the changes occurring in lipid metabolism from the diet to the meat FA profile.

## Materials and Methods

All the experimental procedures performed in this trial were approved by the Animal Ethics Committee of the CITA Research Centre (CEEA, 2017-07), in compliance with the guidelines of Directive 2010/63/EU of the European Parliament and of the Council of 22 September on the protection of animals used for experimental purposes.

### Animal Management and Experimental Design

The study was conducted during autumn 2020 at the CITA facilities (41°3′ N, 0°47′ W and 216 m above sea level) in Zaragoza, Spain. After weaning, 26 male Rasa Aragonesa lambs were randomly separated into three homogeneous groups considering their weaning age (30 ± 2.0 days) and weight (14.0 ± 0.49 kg). Lambs were individually penned indoors for 40 days until slaughter. Each group received a pelleted concentrate with a different level of sainfoin inclusion: a cereal-based concentrate without sainfoin (0SF), a concentrate with 20% sainfoin (20SF), and a concentrate with 40% sainfoin (40SF). Lambs had free access to concentrates, water, and minerals. The sainfoin used in the 20SF and 40SF concentrates was cut at the flowering stage in spring 2019, dehydrated, and kept pelleted until early autumn, when the sainfoin pellets were ground and introduced into the concentrates (3.5 mm-diameter pellets). The pelleted concentrates were formulated to be isoenergetic and isoproteic (Table 1).

**Table 1:** Ingredients and Chemical and Fatty Acid (FA) Composition, Mean ± Standard Deviation, of the Experimental Diets

	diets^a^		
	0SF	20SF	40SF
ingredients, g/kg dry matter (DM)			
barley	310	252	50
corn	250	189	250
wheat	50	50	102
gluten feed	60	60	130
soybean meal 47%	173	138	159
bran	25	81	0
palm oil	10	10	15
calcium carbonate	15	13	4
sodium chloride	5	5	5
premix vitamin 0.2%	2	2	2
sainfoin pellet	0	200	400
straw	100	0	0
chemical composition, g/kg DM			
dry matter, g/kg as fed	905 ± 2.5	904 ± 4.5	903 ± 4.2
crude protein	174 ± 4.3	175 ± 6.5	173 ± 5.2
ether extract	32.6 ± 3.25	35.7 ± 3.60	38.0 ± 3.44
ash	75.2 ± 2.51	70.5 ± 2.06	78.5 ± 5.48
starch	426 ± 6.9	360 ± 13.8	296 ± 9.6
NDF	263 ± 20.9	292 ± 12.1	355 ± 16.4
ADF	129 ± 9.1	168 ± 6.5	249 ± 10.4
ADL	17.0 ± 3.25	34.2 ± 3.17	59.6 ± 4.37
gross energy, MJ/kg DM	18.1 ± 1.37	18.4 ± 1.25	18.4 ± 0.94
proanthocyanidins (PACs)^b^			
total	1.32 ± 0.527	3.04 ± 0.448	5.23 ± 0.550
extractable	0.41 ± 0.152	0.50 ± 0.169	0.75 ± 0.132
protein bound	0.77 ± 0.529	2.07 ± 0.373	3.67 ± 0.508
fiber bound	0.15 ± 0.115	0.47 ± 0.138	0.80 ± 0.118
delphinidin/cyanidin ratio	51:49 ± 1.56	68:32 ± 0.65	71:29 ± 1.06
total fatty acids (FAs), mg/g DM	44.6 ± 1.13	45.9 ± 1.39	46.3 ± 1.97
individual FA, g/100 g total FA			
C12:0	0.08 ± 0.017	0.07 ± 0.011	0.11 ± 0.027
C14:0	0.50 ± 0.022	0.51 ± 0.014	0.61 ± 0.022
C15:0	0.06 ± 0.011	0.08 ± 0.009	0.07 ± 0.008
C16:0	27.6 ± 0.23	28.0 ± 0.18	30.0 ± 0.30
C16:1 c9	0.20 ± 0.069	0.22 ± 0.055	0.27 ± 0.061
C18:0	7.34 ± 0.286	6.98 ± 0.270	7.42 ± 0.339
C18:1 c9	24.6 ± 0.27	24.1 ± 0.32	26.8 ± 0.89
C18:1 c11	0.28 ± 0.205	0.26 ± 0.176	0.28 ± 0.141
C18:2 n-6	36.8 ± 0.25	35.0 ± 0.32	27.8 ± 0.58
C18:3 n-3	2.49 ± 0.137	4.77 ± 0.193	6.71 ± 0.444
SFA^c^	35.6 ± 0.41	35.6 ± 0.39	38.2 ± 0.53
MUFA^d^	25.1 ± 0.18	24.6 ± 0.24	27.3 ± 0.82
PUFA^e^	39.3 ± 0.35	39.8 ± 0.29	34.5 ± 0.57

a 0SF, 0% sainfoin; 20SF, 20% sainfoin; 40SF, 40% sainfoin in the finishing concentrate.

b g eq of sainfoin PAC/kg DM.

c Saturated FA.

d Monounsaturated FA.

e Polyunsaturated FA.

### Sampling Procedures and Slaughter

Composite samples of the concentrates were obtained weekly per animal to determine the chemical and FA composition. At day 40, without prior fasting, the lambs were slaughtered in the CITA experimental slaughterhouse adjacent to the lamb housing facilities. Lambs were stunned by a captive bolt pistol and exsanguinated, using standard commercial procedures and according to Council Regulation (EC) no. 1099/2009. Blood samples were collected from the jugular vein of lambs during exsanguination, kept in tubes containing heparin (Vaccuette, Spain), and immediately centrifuged (3000*g* for 15 min at 4 °C) and stored at −20 °C. Ruminal digesta was extracted (nonfiltered), kept in flasks, freeze-dried, and preserved at −20 °C. The longissimus thoracis et lumborum muscles corresponding to the right side of the carcass, after 24h post-mortem at 4 °C, were excised and sliced between the fourth and sixth lumbar vertebrae to study the intramuscular fat (IMF), cholesterol concentrations, and FA profile of meat. All samples were freeze-dried (Lyobeta 25, Azbil Telstar, Japan) and kept at −20 °C.

### Chemical Analyses

All chemical composition analyses of the concentrates were run in triplicate. The techniques used for the chemical analyses of the concentrates are detailed by Baila et al.^16^ The total starch content of the concentrates was measured with the commercial kit K-TSTA-100A (Neogen Corporation, Lansing, MI, USA) following the amyloglucosidase/α-amylase method.

The dry matter (DM) and IMF of meat were measured by using NIRs (FoodScanTM2, Foss Analytics, Hilleroed, Denmark), and the amount of cholesterol in the meat was determined following the method of Bertolín et al.^17^ using an Acquity UPLC H-Class liquid chromatograph (Waters, Mildford, KA, USA) with a silica-based bonded phase column (Acquity UPLC HSS T3, 150 mm × 2.1 mm × 1.8 μm, Waters), an absorbance detector (Acquity UPLC Photodiode Array PDA eλ Detector, Waters), and a fluorescence detector (2475 Multi λ Fluorescence Detector, Waters) and controlled with Empower 3 software (Waters, Mildford, KA, USA). The absorbance of cholesterol was measured at 220 nm.

Evaluation of the FA profile of concentrates, plasma, and meat samples was performed at the CITA (Spain). The FA profile of the concentrates was analyzed using 500 mg of lyophilized samples following the method described by Rufino-Moya et al.^12^ For plasma and meat FA profiles, 2 mL and 500 mg of lyophilized samples, respectively, were extracted according to Lee et al.^18^ Afterward, all the samples were methylated as FA methyl esters using 4 mL of 0.5 M CH_3_ONa/CH_3_OH solution followed by 4 mL of acetyl chloride/CH_3_OH (1:10, v:v) and extracted in 3 mL of heptane. After, the FA concentration was determined in a Bruker Scion 436-GC gas chromatograph (Bruker, Billerica, MA, USA) with a flame ionization detector equipped with a CP-8400 autosampler and an SP-2560 capillary column (100 m × 0.25 mm ID × 0.20 μm for concentrate samples and 200 m × 0.25 mm ID × 0.20 μm for plasma and meat samples; Sigma-Aldrich, Saint Louis, MO, USA). The technical specifications of the chromatographic conditions followed for the FA analyses of the concentrates can be found in detail in the work of Rufino-Moya et al.^12^ For plasma and meat, the oven temperature was 70 °C for 1 min followed by 5 °C/min for 2 min to 225 °C maintained for 17 min with a total time of 80 min. The injector and detector temperatures were maintained at 260 and 250 °C, respectively. Identification of the FAs of concentrates, plasma, and meat was performed with standard FA mixtures GLC-532, GLC-401, GLC-643, and GLC-642 (Nu-Chek Prep, Inc., Elysian, MN, USA) and compared with the retention times described in the literature.^19−21^ The quantification was performed as described in ISO 12966-4:2015 and expressed as g of FA per 100 g of total FA. Total FA concentration was expressed as mg of FA per g of sample using C19:0 (methyl nonadecanoate N-19-M from Nu-Chek Prep, Inc., Elysian, MN, USA) as the internal standard for concentrates and plasma and C23:0 (methyl tricosanoate N-23-M from Nu-Chek Prep, Inc., Elysian, MN, USA) for meat samples.

The analyses involving the FA determination of ruminal digesta were performed at the Laboratory of Animal Production and Nutrition of the Faculty of Veterinary Medicine, University of Lisbon (Portugal) as described in the work of Alves et al.^22^ Briefly, freeze-dried rumen concentrations (250 mg) were directly transesterified according to the method described by Alves et al.^23^ Methyl nonadecanoate (C19:0) (internal standard) was used for quantification by gas chromatography with flame ionization detection (GC-FID) using a Shimadzu GC 2010-Plus (Shimadzu, Kyoto, Japan) equipped with a SP-2560 (100 m × 0.25 mm × 0.20 μm film thickness, Supelco, Bellefonte, PA, USA) capillary column with the chromatographic conditions described by Alves et al.^24^ The FA determinations were performed by comparison with ruminal chromatograms of Alves et al.^23^ and Alves and Bessa.^25^ Calculations estimating the biohydrogenation extent of C18 dietary FAs in rumen provide an estimation of the degree of ruminal BH to which the main C18 dietary FAs have been subjected, whereas the completeness (%) in the rumen reflects an estimation of the BH extent that has occurred, considering the maximum BH that could be achieved if the entire dietary FAs were completely biohydrogenated.^22^ The sums, ratios, and Δ^9^-desaturation ratios relative to the FA profiles are detailed in the work of Baila et al.^26^

### Statistical Analyses

Data were analyzed with SAS statistical software (v.9.3; SAS Inst. Inc., Cary, NC, USA), considering the animal as the experimental unit. The FA profiles of ruminal digesta, plasma, and meat were analyzed using analysis of variance with a mixed model (MIXED procedure) and the diet (0SF, 20SF, and 40SF) as a fixed effect. When significant, the group statement was included in the model to adjust the variance heterogeneity. The degrees of freedom were adjusted with the Kenward–Roger correction. The least-squares means and their associated standard errors were obtained, and Tukey’s correction was used for pairwise comparisons. The effects were considered significant at *P* < 0.05.

## Results

### FA Composition of Ruminal Digesta

The main differences in the FA profile and the BH extent and completeness of the ruminal concentration due to the diets are listed in Table 2. The diet did not affect the total FA content of ruminal digesta (*P* > 0.05). The total SFA percentage was similar among diets (*P* > 0.05), but the diet had an effect on C16:0, C20:0, C22:0, C24:0, and C28:0 (*P* < 0.001), with higher percentages in 40SF lambs than in their counterparts, except for C20:0, which increased with the level of sainfoin inclusion. The percentage of total branched-chain FA (BCFA) in rumen was affected by the diet (*P* < 0.01), being greater in 0SF than in 20SF lambs. The total percentage of *iso*-BCFA did not differ among the diets (*P* > 0.05), whereas the percentage of total *anteiso-*BCFA was higher in 0SF than in 20SF and 40SF (*P* < 0.05).

**Table 2:** Effect of the Diet on the Fatty Acid (FA) Profile (% of Total FA Identified) and C18 Rumen Biohydrogenation Extent and Completeness (%) of the Ruminal Digesta of Lambs^o^

	diets^a^			
	0SF	20SF	40SF	*P*-value
total fatty acids (FAs), mg FA/g dry matter	43.5 ± 1.24	47.3 ± 1.31	43.6 ± 1.24	0.08
individual FA				
SFA^b^	40.7 ± 2.97	48.1 ± 3.15	45.9 ± 2.97	0.23
C12:0	0.12 ± 0.016	0.12 ± 0.016	0.15 ± 0.016	0.38
C13:0	0.05 ± 0.011	0.06 ± 0.012	0.04 ± 0.011	0.55
C14:0	0.68 ± 0.055	0.62 ± 0.058	0.69 ± 0.055	0.62
C15:0	0.36 ± 0.034	0.29 ± 0.037	0.37 ± 0.034	0.25
C16:0	24.9^b^ ± 0.34	24.5^b^ ± 0.36	29.3^a^ ± 0.34	<0.001
C17:0	0.27 ± 0.017	0.27 ± 0.019	0.27 ± 0.017	0.99
C18:0	13.3 ± 3.06	21.1 ± 3.24	13.7 ± 3.06	0.17
C20:0	0.35^c^ ± 0.013	0.41^b^ ± 0.014	0.46^a^ ± 0.013	<0.001
C21:0	0.02 ± 0.008	0.02 ± 0.008	0.01 ± 0.008	0.67
C22:0	0.24^b^ ± 0.007	0.26^b^ ± 0.007	0.30^a^ ± 0.007	<0.001
C23:0	0.14 ± 0.030	0.10 ± 0.009	0.08 ± 0.019	0.26
C24:0	0.25^b^ ± 0.022	0.29^b^ ± 0.017	0.36^a^ ± 0.008	<0.001
C26:0	0.09 ± 0.00 5	0.11 ± 0.009	0.13 ± 0.016	0.14
C28:0	0.01^b^ ± 0.010	0.04^b^ ± 0.010	0.12^a^ ± 0.010	<0.001
BCFA^c^	1.84^a^ ± 0.137	1.22^b^ ± 0.145	1.36^ab^ ± 0.137	0.01
*iso*-BCFA	0.57 ± 0.080	0.44 ± 0.085	0.54 ± 0.080	0.52
*iso*-C13:0	0.007^b^ ± 0.0062	0.004^b^ ± 0.0065	0.031^a^ ± 0.0062	0.009
*iso*-C14:0	0.13 ± 0.014	0.09 ± 0.015	0.10 ± 0.014	0.21
*iso*-C15:0	0.20 ± 0.046	0.13 ± 0.017	0.20 ± 0.034	0.12
*iso*-C16:0	0.19 ± 0.047	0.20 ± 0.050	0.15 ± 0.047	0.77
*iso*-C17:0	0.04 ± 0.011	0.03 ± 0.011	0.05 ± 0.011	0.39
*anteiso*-BCFA	1.27^a^ ± 0.073	0.78^b^ ± 0.077	0.82^b^ ± 0.073	<0.001
*anteiso*-C15:0	1.02^a^ ± 0.065	0.64^b^ ± 0.068	0.73^b^ ± 0.065	<0.01
*anteiso*-C17:0	0.24^a^ ± 0.034	0.15^ab^ ± 0.036	0.09^b^ ± 0.034	0.02
MUFA^d^	35.7 ± 2.19	29.1 ± 2.33	33.9 ± 2.19	0.13
*cis*-MUFA	13.9^b^ ± 0.82	14.8^ab^ ± 0.87	17.4^a^ ± 0.82	0.02
C16:1 c7/t3^e^	0.11^a^ ± 0.026	0.07^ab^ ± 0.023	0.04^b^ ± 0.010	0.02
C16:1 c9	0.12 ± 0.013	0.11 ± 0.014	0.10 ± 0.013	0.45
C18:1 c9^f^	12.6^b^ ± 0.83	13.2^ab^ ± 0.88	15.8^a^ ± 0.83	0.03
C18:1 c11	0.90 ± 0.062	0.83 ± 0.065	0.92 ± 0.062	0.54
C18:1 c12	0.02 ± 0.014	0.00 ± 0.015	0.02 ± 0.014	0.57
C18:1 t16/c14^g^	0.03^b^ ± 0.014	0.12^a^ ± 0.028	0.22^a^ ± 0.061	0.002
C18:1 c15	0.13^b^ ± 0.054	0.45^a^ ± 0.117	0.24^b^ ± 0.032	0.047
C18:1 c16	0.01^b^ ± 0.002	0.02^ab^ ± 0.009	0.04^a^ ± 0.009	<0.001
*trans*-MUFA	21.9^a^ ± 1.8	14.5^b^ ± 1.91	16.7^ab^ ± 1.8	0.03
C18:1 t6/t7/t8^h^	0.80 ± 0.259	1.43 ± 0.124	1.20 ± 0.117	0.09
C18:1 t9	0.41 ± 0.155	0.80 ± 0.081	0.62 ± 0.056	0.08
C18:1 t10	19.5^a^ ± 1.88	10.3^b^ ± 2.00	9.24^b^ ± 1.883	0.01
C18:1 t11	0.47^b^ ± 0.108	1.14^b^ ± 0.345	4.70^a^ ± 0.6080	<0.001
C18:1 t12	0.68^a^ ± 0.066	0.39^b^ ± 0.070	0.41^b^ ± 0.066	0.008
C18:1 t15	0.09^b^ ± 0.051	0.30^a^ ± 0.054	0.32^a^ ± 0.051	0.008
PUFA^i^	17.3^a^ ± 1.03	16.9^a^ ± 1.09	12.8^b^ ± 1.03	0.009
C18:2 n-6	15.6^a^ ± 0.87	14.3^a^ ± 0.93	9.97^b^ ± 0.874	<0.001
C18:2 t9,c12	0.02 ± 0.010	0.06 ± 0.022	0.11 ± 0.037	0.05
C18:2 t11,c15/t10,c15^j^	0.35^b^ ± 0.035	0.59^ab^ ± 0.146	0.69^a^ ± 0.129	0.03
C18:3 n-3	1.25^b^ ± 0.105	1.76^a^ ± 0.112	1.78^a^ ± 0.105	0.002
C18:3 c9,t11,c15	0.01^b^ ± 0.005	0.11^a^ ± 0.005	0.16^a^ ± 0.020	<0.001
CLA^k^	0.10 ± 0.017	0.12 ± 0.018	0.08 ± 0.017	0.30
CLA c9,t11	0.07 ± 0.015	0.07 ± 0.016	0.07 ± 0.015	0.99
CLA t10,c12	0.03^a^ ± 0.007	0.04^a^ ± 0.08	0.00^b^ ± 0.007	0.002
CLA t11,c13	0.00 ± 0.004	0.01 ± 0.005	0.01 ± 0.005	0.20
oxo-FA	2.41 ± 0.283	1.97 ± 0.196	3.61 ± 0.649	0.05
C18:0 oxo-12/-13^l^	1.41^a^ ± 0.142	0.94^b^ ± 0.117	2.07^a^ ± 0.416	0.009
C18:0 oxo-10	1.00 ± 0.219	1.03 ± 0.232	1.54 ± 0.219	0.17
C18:1 t10/C18:1 t11	54.5^a^ ± 18.83	13.2^b^ ± 2.80	1.98^c^ ± 0.279	<0.001
BI^m^	24.9 ± 2.10	17.7 ± 2.23	21.6 ± 2.10	0.089
C18:1 t10 (% BI)^n^	75.7^a^ ± 4.15	55.8^b^ ± 4.15	40.2^c^ ± 4.15	<0.001
biohydrogenation extent, %				
C18:1 c9	46.5 ± 3.50	43.6 ± 3.71	36.0 ± 3.50	0.12
C18:2 n-6	55.8 ± 2.64	57.9 ± 2.80	61.3 ± 2.64	0.35
C18:3 n-3	47.6^c^ ± 2.36	61.9^b^ ± 2.50	71.4^a^ ± 2.36	<0.001
completeness, %	34.2 ± 6.55	51.9 ± 6.95	37.2 ± 6.55	0.17

a 0SF, 0% sainfoin; 20SF, 20% sainfoin; 40SF, 40% sainfoin in the finishing concentrate.

b Saturated FA.

c Branched-chain FA.

d Monounsaturated FA.

e C16:1 c7 and C16:1 t3 might coelute.

f C18:1 c9 might coelute with the pair C18:1 t13 and t14.

g C18:1 t16 coelutes with C18:1 c14 as a minor isomer.

h C18:1 t6, C18:1 t7, and C18:1 t8 might coelute.

i Polyunsaturated FA.

j C18:2 t11,c15 and C18:2 t10,c15 might coelute.

k Conjugated linoleic acid.

l C18:0 oxo-12 and C18:0 oxo-13 might coelute.

m Biohydrogenation intermediates: all C18 FA except C18:0, C18:1 c9, C18:1 c11, C18:2 n-6, and C18:3 n-3.

n Proportion of BI explained by C18:1 t10.

o The different lowercase letters indicate differences among groups at *P* < 0.05; standard error of means (±).

Although the total ruminal percentage of monounsaturated FA (MUFA) was similar among diets (*P* > 0.05), 0SF lambs had lower percentages of total *cis*-MUFA, C18:1 c9, C18:1 c16, and C18:1 t11 than 40SF lambs and greater percentages of C18:1 t10 and C18:1 t12 than either 20SF or 40SF lambs (*P* < 0.05). Concerning the percentage of total *trans*-MUFA in the rumen, the 20SF diet reduced their values when compared with that obtained with the 0SF diet (*P* < 0.05).

The diet changed the percentages of total PUFA, C18:2 n-6, and CLA t10,c12 (*P* < 0.01), showing the rumen values of 40SF lambs lower than those of their counterparts. The percentages of C18:3 n-3 and C18:3 c9,t11,c15 were lower in the rumen of 0SF lambs than those of their counterparts (*P* < 0.05), which presented similar percentages (*P* > 0.05). The ruminal C18:2 t11,c15/t10,c15 percentage was lower in 0SF compared with 40SF (*P* < 0.05), whereas 20SF lambs presented intermediate values (*P* > 0.05). The effect of the diet was also significant for the percentage of C18:0 oxo-12/13 (*P* < 0.01), with lower values detected in 20SF lambs than in their counterparts.

Both the C18:1 t10/C18:1 t11 ratio and the proportion of biohydrogenation intermediates (BI) explained by the presence of C18:1 t10 were affected by the diet (*P* < 0.001), decreasing as the level of sainfoin increased in the diet, but the BI remained unaffected (*P* > 0.05). The diet did not affect the BH extent (completeness) (*P* > 0.05) but affected the BH extent of C18:3 n-3, which increased with the level of inclusion of sainfoin (*P* < 0.001).

### FA Composition of Plasma

The total FA content in plasma was affected by the diet (Table 3), showing higher values in 0SF, intermediate in 20SF, and low in 40SF lambs (*P* < 0.05). The diet did not affect the total SFA percentage or those of individual SFAs (*P* > 0.05), except for C15:0 (*P* < 0.001), which was greater in 40SF.

**Table 3:** Effect of the Diet on the Total Fatty Acid (FA) Concentration and FA Profile (% of Total FA Identified) of Plasma Lambs^e^

	diets^a^			
	0SF	20SF	40SF	*P*-value
total fatty acids (FAs), mg FA/mL plasma	2.68^a^ ± 0.102	2.67^ab^ ± 0.108	2.31^b^ ± 0.102	0.004
individual FA				
SFA^b^	78.9 ± 0.98	80.1 ± 1.03	80.0 ± 0.98	0.66
C12:0	1.37 ± 0.086	1.46 ± 0.092	0.71 ± 0.034	0.05
C14:0	0.71 ± 0.034	0.77 ± 0.036	0.71 ± 0.034	0.34
C15:0	0.17^b^ ± 0.017	0.14^b^ ± 0.018	0.25^a^ ± 0.017	<0.001
C16:0	41.6 ± 0.42	41.3 ± 0.44	41.1 ± 0.42	0.76
C17:0	0.37 ± 0.034	0.40 ± 0.036	0.44 ± 0.034	0.31
C18:0	34.6 ± 0.65	35.9 ± 0.69	35.6 ± 0.65	0.37
C20:0	0.07 ± 0.016	0.06 ± 0.012	0.11 ± 0.031	0.32
C22:0	0.04 ± 0.012	0.06 ± 0.012	0.03 ± 0.012	0.18
C24:0	0.04 ± 0.007	0.04 ± 0.008	0.03 ± 0.007	0.34
MUFA^c^	9.30 ± 0.571	8.94 ± 0.605	8.83 ± 0.571	0.84
*cis*-MUFA	7.01 ± 0.402	7.70 ± 0.427	7.10 ± 0.402	0.45
C16:1 c9	0.35^a^ ± 0.033	0.26^ab^ ± 0.034	0.15^b^ ± 0.033	<0.001
C18:1 c9	5.96 ± 0.360	6.76 ± 0.382	6.36 ± 0.360	0.33
C18:1 c11	0.57 ± 0.044	0.57 ± 0.047	0.50 ± 0.044	0.37
C20:1 c11	0.03 ± 0.013	0.05 ± 0.014	0.03 ± 0.013	0.67
C22:1 c13	0.04^ab^ ± 0.014	0.02^b^ ± 0.005	0.05^a^ ± 0.007	0.01
C24:1 c15	0.05^a^ ± 0.006	0.04^ab^ ± 0.015	0.02^b^ ± 0.006	0.005
*trans*-MUFA	2.29 ± 0.308	1.24 ± 0.326	1.73 ± 0.308	0.08
C18:1 t10	1.59 ± 0.212	0.96 ± 0.225	1.26 ± 0.212	0.15
C18:1 t11	0.70^a^ ± 0.100	0.28^b^ ± 0.106	0.47^ab^ ± 0.100	0.02
PUFA^d^	11.8 ± 0.57	11.0 ± 0.61	11.2 ± 0.57	0.60
PUFA n-6	11.3 ± 0.56	10.2 ± 0.59	10.2 ± 0.56	0.28
C18:2 n-6	10.1 ± 0.53	9.10 ± 0.567	9.43 ± 0.53	0.42
C18:3 n-6	0.14^a^ ± 0.021	0.08^ab^ ± 0.022	0.07^b^ ± 0.021	0.04
C20:2 n-6	0.05^ab^ ± 0.008	0.08^a^ ± 0.023	0.02^b^ ± 0.007	0.008
C20:3 n-6	0.05^b^ ± 0.012	0.09^a^ ± 0.013	0.08^ab^ ± 0.013	0.046
C20:4 n-6	0.95^a^ ± 0.053	0.79^ab^ ± 0.056	0.72^b^ ± 0.057	0.02
PUFA n-3	0.43^b^ ± 0.065	0.80^a^ ± 0.069	0.93^a^ ± 0.065	<0.001
C18:3 n-3	0.27^c^ ± 0.036	0.41^b^ ± 0.038	0.60^a^ ± 0.036	<0.001
C20:5 n-3	0.09 ± 0.025	0.10 ± 0.027	0.07 ± 0.027	0.77
C22:5 n-3	0.01^b^± 0.020	0.16^a^ ± 0.022	0.18^a^ ± 0.022	<0.001
C22:6 n-3	0.05^b^ ± 0.017	0.12^a^ ± 0.018	0.10^ab^ ± 0.017	0.02
C18:1 t10/C18:1 t1	2.55 ± 0.313	5.53 ± 1.638	5.96 ± 3.321	0.15

a 0SF, 0% sainfoin; 20SF, 20% sainfoin; 40SF, 40% sainfoin in the finishing concentrate.

b Saturated FA.

c Monounsaturated FA.

d Polyunsaturated FA.

e The different lowercase letters indicate differences among groups at *P* < 0.05; standard error of means (±).

The total percentage of MUFA and *cis*- and *trans*-MUFA (*P* > 0.05) showed no effect of the diet but the percentage of four individual MUFAs showed differences among diets (*P* < 0.05). Thus, the plasma of 0SF lambs had greater percentages of C16:1 c9 and C24:1 c15 compared with that of 40SF lambs and a greater percentage of C18:1 t11 compared with that of 20SF, while the C22:1 c13 percentage was greater in 40SF lambs than in 20SF lambs (*P* < 0.05).

The percentages of PUFA, PUFA n-6, and its major FA, C18:2 n-6, showed no changes among diets (*P* > 0.05) despite the changes observed on the percentages of C18:3 n-6, C20:2 n-6, C20:3 n-6, and C20:4 n-6 due to the diet (*P* < 0.05). The plasma of 0SF lambs had greater percentages of C18:3 n-6 and C20:4 n-6 than that of 40SF lambs and lower percentage of C20:3 n-6 than that of 20SF lambs, while the percentage of C20:2 n-6 was greater in 20SF than in 40SF lambs.

The diet affected the total percentage of PUFA n-3 (*P* < 0.001), C18:3 n-3 (*P* < 0.001), C22:5 n-3 (*P* < 0.001), and C22:6 n-3 (*P* < 0.05), with lower percentages in the plasma of 0SF lambs than in their counterparts, which presented similar percentages between them (*P* > 0.05), except for C18:3 n-3, the percentage of which increased with the higher inclusion of sainfoin (*P* < 0.001).

### FA Composition of the Longissimus Lumborum Muscle

The diet had no effect on the DM, IMF, and cholesterol (*P* > 0.05, Table 4) percentages of meat but affected the percentage of total FA (*P* < 0.05), which was higher in 0SF than in 40SF, whereas 20SF presented intermediate values.

**Table 4:** Effect of the Diet on the Intramuscular Fat (IMF), Cholesterol, Total Fatty Acid (FA) Concentration (g/100 g of Fresh Muscle), and FA Profile (% of Total FA Identified) of the Longissimus Lumborum Muscle of Lambs^m^

	diets^a^			
	0SF	20SF	40SF	*P-*value
dry matter	22.1 ± 0.05	22.0 ± 0.05	22.3 ± 0.05	0.66
IMF	3.09 ± 0.151	2.99 ± 0.160	2.98 ± 0.151	0.87
cholesterol	0.136 ± 0.0038	0.136 ± 0.0040	0.140 ± 0.0038	0.74
total fatty acids (FAs)	0.93^a^ ± 0.041	0.84^ab^ ± 0.045	0.79^b^ ± 0.041	0.046
individual FA				
SFA^b^	44.7 ± 0.47	44.6 ± 0.46	45.6 ± 0.46	0.28
C10:0	0.49 ± 0.050	0.52 ± 0.053	0.46 ± 0.053	0.72
C11:0	0.02^a^ ± 0.001	0.01^b^ ± 0.002	0.03^a^ ± 0.003	0.005
C12:0	0.34 ± 0.029	0.36 ± 0.029	0.40 ± 0.029	0.34
C13:0	0.08 ± 0.011	0.06 ± 0.012	0.06 ± 0.011	0.25
C14:0	2.51 ± 0.142	2.31 ± 0.142	2.37 ± 0.141	0.59
C15:0	0.72^a^ ± 0.030	0.57^b^ ± 0.032	0.49^b^ ± 0.030	<0.001
C16:0	21.1 ± 0.35	20.4 ± 0.35	21.3 ± 0.35	0.15
C17:0	0.91^a^ ± 0.037	0.87^ab^ ± 0.039	0.77^b^ ± 0.037	0.04
C18:0	10.9 ± 0.22	11.3 ± 0.23	11.4 ± 0.22	0.24
C20:0	0.09 ± 0.007	0.07 ± 0.006	0.11 ± 0.028	0.06
C21:0	0.02 ± 0.011	0.03 ± 0.012	0.04 ± 0.011	0.39
C22:0	0.01 ± 0.001	0.02 ± 0.004	0.03 ± 0.016	0.06
C24:0	0.02 ± 0.006	0.03 ± 0.006	0.04 ± 0.006	0.10
BCFA^c^	2.11a ± 0.071	1.80^b^ ± 0.070	1.47^c^ ± 0.069	<0.001
*iso*-BCFA	1.52^a^ ± 0.046	1.35^b^ ± 0.045	1.12^c^ ± 0.044	<0.001
*iso*-C15:0	0.77 ± 0.037	0.75 ± 0.037	0.66 ± 0.037	0.14
*iso*-C16:0	0.09^a^ ± 0.005	0.05^b^ ± 0.005	0.05^b^ ± 0.005	<0.001
*iso*-C17:0	0.34^a^ ± 0.017	0.26^b^ ± 0.018	0.24^b^ ± 0.017	<0.001
*iso*-C18:0	0.28 ± 0.034	0.30 ± 0.036	0.20 ± 0.034	0.12
*anteiso*-BCFA	0.60^a^ ± 0.037	0.46^b^ ± 0.040	0.35^b^ ± 0.037	<0.001
*anteiso*-C13:0	0.20^a^ ± 0.024	0.17^ab^ ± 0.024	0.11^b^ ± 0.023	0.04
*anteiso*-C15:0	0.09 ± 0.007	0.07 ± 0.021	0.07 ± 0.009	0.19
*anteiso*-C17:0	0.32^a^ ± 0.014	0.22^b^ ± 0.015	0.18^b^ ± 0.014	<0.001
MUFA^d^	28.4 ± 0.65	28.6 ± 0.64	28.7 ± 0.63	0.96
*cis*-MUFA	24.3 ± 0.68	25.0 ± 0.67	25.4 ± 0.66	0.52
C16:1 c9	1.29 ± 0.077	1.23 ± 0.077	1.20 ± 0.076	0.70
C16:1 c11	0.01 ± 0.004	0.02 ± 0.003	0.03 ± 0.008	0.30
C16:1 c12	0.01 ± 0.004	0.03 ± 0.004	0.02 ± 0.004	0.08
C18:1 c6/c8^e^	0.15 ± 0.018	0.12 ± 0.019	0.15 ± 0.018	0.40
C18:1 c9	20.8 ± 0.66	21.1 ± 0.70	21.4 ± 0.66	0.82
C18:1 c11	1.73^a^ ± 0.050	1.61^ab^ ± 0.050	1.50^b^ ± 0.049	0.02
C18:1 c12	0.05^b^ ± 0.011	0.05^b^ ± 0.015	0.15^a^ ± 0.026	0.007
C18:1 c13	0.01 ± 0.007	0.04 ± 0.007	0.03 ± 0.007	0.07
C18:1 c14	0.04^a^ ± 0.005	0.01^b^ ± 0.005	0.02^b^ ± 0.005	0.01
C18:1 c15	0.02 ± 0.007	0.04 ± 0.007	0.03 ± 0.007	0.15
C20:1 c11	0.11^b^ ± 0.007	0.15^a^ ± 0.014	0.06^c^ ± 0.011	<0.001
C22:1 c13	0.02 ± 0.004	0.02 ± 0.004	0.01 ± 0.004	0.16
C24:1 c15	0.08^a^ ± 0.013	0.03^b^ ± 0.010	0.02^b^ ± 0.005	<0.001
*trans*-MUFA	3.41^a^ ± 0.246	2.69^ab^ ± 0.261	2.50^b^ ± 0.246	0.04
C16:1 t9	0.06^c^ ± 0.005	0.07^b^ ± 0.006	0.12^a^ ± 0.014	0.002
C16:1 t10	0.02^ab^ ± 0.004	0.01^b^ ± 0.001	0.02^a^ ± 0.003	0.007
C17:1 t9	0.11^a^ ± 0.011	0.03^ab^ ± 0.011	0.06^b^ ± 0.011	0.01
C18:1 t5	0.01 ± 0.002	0.005 ± 0.0017	0.01 ± 0.002	0.15
C18:1 t6/t8^f^	0.04 ± 0.010	0.06 ± 0.010	0.05 ± 0.010	0.32
C18:1 t9	0.10^b^ ± 0.012	0.15^a^ ± 0.013	0.16^a^ ± 0.012	0.003
C18:1 t10	2.27^a^ ± 0.205	1.60^ab^ ± 0.217	1.32^b^ ± 0.205	0.01
C18:1 t11	0.73 ± 0.074	0.64 ± 0.078	0.66 ± 0.074	0.71
C18:1 t12	0.07^b^ ± 0.008	0.07^b^ ± 0.008	0.10^a^ ± 0.008	0.001
PUFA^g^	23.2 ± 0.34	23.7 ± 0.97	23.0 ± 0.63	0.78
CLA^h^	0.33 ± 0.026	0.33 ± 0.028	0.36 ± 0.026	0.64
CLA t7,c9	0.02 ± 0.010	0.01 ± 0.003	0.01 ± 0.002	0.41
CLA c9,t11	0.16^b^ ± 0.014	0.14^b^ ± 0.015	0.22^a^ ± 0.014	0.003
CLA t9,c11	0.10 ± 0.011	0.10 ± 0.011	0.06 ± 0.011	0.05
CLA t10,c12	0.01 ± 0.002	0.01 ± 0.002	0.01 ± 0.002	0.97
PUFA n-6	18.5 ± 0.70	18.1 ± 0.69	16.7 ± 0.68	0.19
C18:2 n-6	12.8 ± 0.29	12.9 ± 0.9	11.9 ± 0.48	0.26
C18:3 n-6	0.12^a^ ± 0.007	0.12^a^ ± 0.008	0.09^b^ ± 0.093	0.02
C20:2 n-6	0.15 ± 0.005	0.15 ± 0.018	0.13 ± 0.012	0.26
C20:3 n-6	0.44 ± 0.013	0.43 ± 0.013	0.42 ± 0.013	0.37
C20:4 n-6	4.32 ± 0.131	4.20 ± 0.131	3.97 ± 0.129	0.19
C22:4 n-6	0.39^a^ ± 0.017	0.35^a^ ± 0.017	0.28^b^ ± 0.016	0.002
C22:5 n-6	0.08 ± 0.014	0.09 ± 0.005	0.07 ± 0.008	0.13
PUFA n-3	3.30^c^ ± 0.105	4.00^b^ ± 0.112	4.69^a^ ± 0.105	<0.001
C18:3 n-3	0.82^c^ ± 0.036	1.17^b^ ± 0.038	1.64^a^ ± 0.036	<0.001
C20:3 n-3	0.01 ± 0.004	0.02 ± 0.004	0.02 ± 0.004	0.14
C20:5 n-3	0.74^b^ ± 0.042	0.91^a^ ± 0.044	1.02^a^ ± 0.042	<0.001
C22:5 n-3	1.30 ± 0.047	1.38 ± 0.047	1.38 ± 0.046	0.37
C22:6 n-3	0.47^b^ ± 0.020	0.52^ab^ ± 0.018	0.60^a^ ± 0.042	0.03
C18:1 t10/C18:1 t11	3.30 ± 0.411	2.86 ± 0.436	1.98 ± 0.411	0.09
PUFA/SFA	0.52 ± 0.010	0.53 ± 0.025	0.50 ± 0.018	0.60
PUFA n-6/PUFA n-3	5.44^a^ ± 0.206	4.57^b^ ± 0.218	3.70^c^ ± 0.206	<0.001
AI index^i^	0.62 ± 0.022	0.60 ± 0.022	0.63 ± 0.021	0.55
TI index^j^	1.02 ± 0.020	0.96 ± 0.020	0.95 ± 0.019	0.07
H:h index^k^	1.73 ± 0.055	1.81 ± 0.054	1.73 ± 0.053	0.44
Δ^9^-desaturase C16 (%)	5.72 ± 0.176	5.68 ± 0.437	5.25 ± 0.211	0.26
Δ^9^-desaturase C18 (%)	65.6 ± 0.83	65.0 ± 0.88	65.2 ± 0.83	0.87
elongase (%)	57.9 ± 0.70	60.1 ± 0.74	59.9 ± 0.70	0.07

a 0SF, 0% sainfoin; 20SF, 20% sainfoin; 40SF, 40% sainfoin in the finishing concentrate.

b Saturated FA.

c Branched-chain FA.

d Monounsaturated FA.

e C18:1 c6 and C18:1 c8 might coelute.

f C18:1 t6 and C18:1 t8 might coelute.

g Polyunsaturated FA.

h Conjugated linoleic acid. ^i^Atherogenicity index.

i Atherogenicity index.

j Thrombogenicity index.

k Hyper-hypocholesterolemic index.

m The different lowercase letters indicate differences among groups at *P* < 0.05; standard error of means (±).

Total SFA percentage and most individual SFA percentages were unaffected by the diet (*P* > 0.05; Table 4), except for those of C15:0 (*P* < 0.001) and C17:0 (*P* < 0.05), which were greater in 0SF than in 40SF lambs, and of C11:0 (*P* < 0.01), with lower values in 20SF than in their counterparts. The diet affected the total concentration of BCFA, *iso*-BCFA, and *anteiso*-BCFA (*P* < 0.001). The percentages of total-BCFA and *iso*-BCFA decreased as the level of sainfoin inclusion increased in the diet, whereas the percentage of total *anteiso*-BCFA decreased due to the presence of sainfoin in the diet. Similarly, percentages of *iso-*C16:0, *iso*-C17:0, total *anteiso*-BCFA, and *anteiso-*C17:0 were greater in 0SF than in their counterparts (*P* < 0.05) regardless of the level of sainfoin inclusion, whereas the percentage of *anteiso-*C13:0 was greater in the 0SF meat than in the 40SF lambs (*P* < 0.05).

The diet did not change the total percentage of MUFA, *cis*-MUFA, or C18:1 c9 in meat (*P* > 0.05; Table 4) but affected the percentage of four minor individual *cis*-MUFAs. The meat of 0SF lambs had a higher C24:1 c14 percentage than those of their counterparts regardless of the proportion of sainfoin and a higher C18:1 c11 percentage only in that of 40SF lambs (*P* < 0.05). The percentages of C18:1 c12 and C20:1 c11 were also affected by the diet (*P* < 0.01 and *P* < 0.001, respectively), with the greatest percentages in 40SF and 20SF lambs, respectively. The diet affected the total percentage of *trans*-MUFA and six of the nine individual FA detected (*P* < 0.05). The meat of 0SF lambs had greater percentages of total *trans*-MUFA, C17:1 t9, and C18:1 t10 than that of the 40SF lambs but had the lowest percentages of C16:1 t9 and C18:1 t9 compared to the meat of their counterparts regardless of the level of inclusion of sainfoin and had a lower percentage of C18:1 t12 than that in 40SF meat (*P* < 0.05). The percentage of C16:1 t10 was lower in the 20SF lambs than in their counterparts (*P* < 0.05).

The diet did not affect the percentage of total PUFA, total CLA, or total PUFA n-6 (*P* > 0.05; Table 4) and only affected the CLA c9,t11 percentage (*P* < 0.01), which was highest in the 40SF meat, and the C18:3 n-6 (*P* < 0.05) and C22:4 n-6 (*P* > 0.01) percentages, which were lowest in 40SF meat. Increasing the level of sainfoin inclusion in the diet increased the percentages of total PUFA n-3 (*P* < 0.001) and individual C18:3 n-3 (*P* < 0.001), C20:5 n-3 (*P* < 0.001), and C22:6 n-3 (*P* < 0.05), with the 40SF meat having higher percentages than those in the 0SF and 20SF meat, except for C20:5 n-3 and C22:6 n-3, which were similar in 20SF and 40SF meat (*P* > 0.05).

The diet did not affect the PUFA/SFA ratio, AI index, TI index, H:h index, Δ^9^-desaturase C16 (%), Δ^9^-desaturase C18 (%), or elongase (%) but affected the PUFA n-6/PUFA n-3 ratio, which decreased as the inclusion of sainfoin in the diet increased (*P* < 0.001; Table 4).

## Discussion

The results concerning feed intake, growth, ruminal fermentation, and carcass traits of the lambs have been reported elsewhere.^15^ Briefly, the 40SF lambs had a greater average daily dry matter (DM) intake (741, 745, and 895 ± 17.8 g DM/d for 0SF, 20SF, and 40SF, respectively; *P* < 0.001). The inclusion of 20 and 40% sainfoin in the concentrate did not show noticeable differences concerning the intake of the individual FAs compared to that of the concentrate without sainfoin (Supplementary Table S1); however, lambs fed the 40SF diet showed a greater total fatty acid (FA) intake, with C18:3 n-3 being the most affected FA because of the inclusion of sainfoin. No differences were found among diets in the final body weight and cold carcass weight. Regarding ruminal parameters, the rumen pH observed in 20SF lambs was lower than that in 40SF (5.87, 5.68, and 6.34 ± 0.164 for 0SF, 20SF, and 40SF, respectively; *P* < 0.05), and the production of total volatile FAs was unaffected by the diet, although both 20SF and 40SF had a higher acetic acid percentage than that in the 0SF group (49.2, 60.6, and 58.5 ± 2.24% for 0SF, 20SF, and 40SF, respectively; *P* < 0.01).

First, it is important to highlight that, in addition to the effect produced by the inclusion of sainfoin in the concentrate, differences in the cereal content among the diets can play an important role in the changes in ruminal BH. To maintain the isoproteic and isoenergetic conditions of the feeds, the inclusion of 20 and 40% sainfoin caused a significant decrease in the cereal content. These differences were especially evident in barley, whose content shows minor variations between 0SF and 20SF (310 and 252 g/kg of DM, respectively) but were much lower in 40SF (50 g/kg DM). Consequently, these differences would have contributed to the effects obtained in the 40SF rumen compared with those of the other diets.

The significant effects on the rumen FA profile showed that the different diets interfered with the ruminal BH and/or with the rumen microbiota. Higher percentages of *anteiso*-BCFA were observed in the rumen of 0SF lambs probably because of the higher concentration of starch in their diet. Fievez et al.^27^ demonstrated that increased productions of *anteiso*-BCFA were linked to increased amylolytic populations. Moreover, although PACs have inhibitory effects on microbial growth and on proteolysis, reducing ruminal concentrations of BCFA,^28^ we suggest that the low PAC content in the sainfoin concentrates compared with that in fresh sainfoin^12,16^ due to the pelleting process could not be enough to justify the changes in BCFA ruminal concentrations.

Regarding the MUFA percentages in the rumen, C18:1 c9 may come from the diet or from the BH of C18:2 n-6.^29^ In this study, a greater C18:1 c9 percentage was observed in the 40SF rumen, which would be related to its greater content in the diet, given the statistically similar BH extents of C18:2 n-6 and C18:1 c9 among diets. Toral et al.^30^ and Huyen et al.^31^ studied sainfoin *in vitro* and in dairy cows fed sainfoin silage and observed a low BH extent and higher C18:1 c9 ruminal percentages coming from the diet. However, because the C18:1 c9 could not be chromatographically resolved from the C18:1 t13/t14 pair, the proportion of C18:1 c9 may also be related to the eventual presence of C18:1 t13 formed from the BH of C18:3 n-3,^32^ which was higher in 40SF meat.

Several of the most important effects produced by sainfoin inclusion in the diet were observed in the *trans*-MUFAs. The study of this group of FA is important because *trans*-MUFAs in the food industry have been phased out due to their potential negative effects on human health so that ruminant-derived products have become one of the main sources of consumption of these types of fats.^32^ The *trans*-FA present in ruminant meat and milk is produced during ruminal BH, and C18:1 t10 and C18:1 t11 are the main FAs formed during this process. High ruminal concentrations of C18:1 t11 have been associated with forage-rich diets, whereas the formation of the C18:1 t10 isomer is linked with concentrate-rich diets.^11^ As the high concentration of C18:1 t10 in products is undesirable, the study of the modification of ruminal BH to enhance the production of C18:1 t11 is a main target in ruminant nutrition because this is converted to CLA c9,t11 in meat through activity of Δ^9^-desaturase, having potential beneficial implications for human health.^33^ The predominance of the t11 or the t10 BH pathway is studied by using the C18:1 t10/C18:1 t11 ratio. It should be noted that the C18:1 t10/C18:1 t11 ratio obtained with the 0SF diet was extraordinarily high compared to the existing literature on lambs fed almost exclusively on cereals;^34^ however, the inclusion of sainfoin in the diet is shown to be able to decrease C18:1 t10 ruminal concentrations, although the concentration of C18:1 t11 and consequently the C18:1 t10/C18:1 t11 ratio only increased in the 40SF group. The reduction in the rumen of approximately 50% of the C18:1 t10 concentration when sainfoin was included in the concentrate suggests a greater presence of the t11 BH pathway in both sainfoin concentrates than in the 0SF meat, which was also supported by the decrease in CLA t10,c12 ruminal percentage when sainfoin was included at 40%. Indeed, this FA is directly derived from the *trans*-10 shifted BH pathway of C18:2 n-6^35^ and has been associated with negative effects on lipid metabolism, specifically with milk fat depression in dairy cows.^36^ The predominance of the t11 BH pathway with the inclusion of 40% sainfoin was confirmed with the increase in C18:1 t11 concentration, which was almost 6-fold higher in 40SF meat than that in 0SF or 20SF meat, and was mainly produced as a result of the t11 BH pathways of C18:3 n-3 and C18:2 n-6 after the hydrogenation of the C18:2 t11,c15 and CLA c9,t11 intermediates, respectively.^11,22^ This result confirms the efficacy of increasing C18:1 t11 as the main isomer formed in the rumen of animals fed forage-rich diets.^23^

As expected, the numerically higher values of BI were negatively related to the values of C18:0 and BH completeness, indicating a more incomplete BH process. However, the differences observed in C18:1 t10 (%BI) showed that as the presence of sainfoin in the concentrate increased, lower proportions of the BI were represented by C18:1 t10, thus supporting the predominance of the t11 pathway over the t10 pathway, as indicated by the differences in the C18:1 t10/C18:1 t11 ratio and the C18:1 t10 values in rumen. However, at this point, it is important to emphasize that the concentrations of C18:1 t10, C18:1 t11, and therefore, the C18:1 t10/C18:1 t11 ratio could also be affected by the differences in starch and NDF content among diets. Both nutrients greatly affect this ratio, and it is well-known that reducing starch and increasing NDF contents of the diet lead to lower concentrations of the C18:1 isomer t10.^11,37,38^

The observed decrease of approximately 40% of C18:2 n-6 in the ruminal content of 40SF lambs compared with those in the rest of the diets could be associated with the 25% lower percentage of this FA in the diet. As C18:2 n-6 is the main FA in the diets and is on average 7-fold more abundant than C18:3 n-3, the lower percentage of C18:2 n-6 in the 40SF diet was reflected in the lower ruminal concentration of total PUFAs. The inclusion of sainfoin in the diet also increased the BH extent of C18:3 n-3, and this effect was greater when sainfoin was included at 40%. It is frequently reported that an increase in C18 PUFA BH occurs when the intake of PUFA is high^39^ as a response of their greater availability and as a defense against the potential toxicity of dietary PUFA to ruminal bacteria.^40^ The higher fiber content of the 40SF concentrate when compared with the rest of the diets could have contributed to the increase in C18:3 n-3 BH extent, producing a longer retention time of the feed in the rumen and a better environment for cellulolytic bacteria, which would cause a greater extension of the BH process.^41^ However, increasing the BH extent of dietary PUFAs is not always positive since the disappearance of PUFAs can reduce their content in the final product. Otherwise, it is important to highlight that in this study, the concentration of C18:3 n-3 found in 40SF rumen was equal to that of 20SF rumen and higher than that in the 0SF group, despite the large BH extent. Increased concentrations of C18:3 n-3 in the ruminal content when using sainfoin have already been found in previous studies.^30,31,42,43^ Nevertheless, this finding is usually associated with the presence of PACs in sainfoin, which can affect the BH of dietary PUFA.^13^ Herein, the lack of C18:3 n-3 BH inhibition may be associated with the low PAC content in sainfoin diets and their low activity. Hence, the higher ruminal C18:3 n-3 concentration in the 20SF and 40SF groups was simply related to a higher availability of this FA in the diet because of the inclusion of sainfoin. Furthermore, this effect was even higher in 40SF probably because of the higher C18:3 n-3 intake observed in this group.

Regarding the FA profile of plasma samples, the effect of sainfoin inclusion on the FA profile was more diluted. Although the changes found for C18:3 n-3 and PUFA n-3 were close to those obtained in the ruminal digesta, the concentrations of MUFA, C18:1 t11, and C18:1 t10 did not reflect the effect obtained in the rumen. Therefore, in this case, plasma samples were not useful to predict what happened during ruminal BH. The lack of a clear link between rumen and plasma FA profile has been previously observed^44,45^ and may be because of the transfer of long-chain FAs to tissues, which depends on numerous factors such as lipid transport and metabolism.^46^

The meat FA profile reflects the final effect of the ruminal BH produced in the diet. The differences in the total amount of FA in meat were not expected in regard to the amount of FA ingested or the rumen results; however, they followed a similar pattern to that observed in the plasma. This may be because the rumen is a direct reflection of the diet, whereas more components of lipid metabolism, such as *de novo* FAs synthesis or even fat mobilization, are involved in the case of plasma or meat, which would explain their greater similarity. The amount of IMF was similar or even higher than that found in light lambs slaughtered at similar weight.^14,47,48^ However, the FA content of meat was found to be very low in all groups compared with the 1.7% of total FA obtained on average in lambs of 28.2 kg BW^49^ and up to 2.6% of FA in meat from heavier lambs (38 kg BW).^50^ Considering that the analysis techniques carried out to determine the IMF and total FA are different and not entirely comparable, this could indicate that a low percentage of the IMF was related to FA, the IMF being composed mostly of phospholipids. Moreover, the FA content was even lower in the meat of lambs fed 40% sainfoin. Despite the differences observed in *cis*-MUFA, C18:2 n-6, PUFA n-6, and total PUFAs between groups in the ruminal FA profile, no changes were found in meat. By contrast, the changes in rumen percentages of BCFAs with sainfoin inclusion were partially reflected in lower percentages of total BCFAs and *anteiso*-BCFAs in meat. In addition, despite no differences in *iso*-BCFAs in rumen, this group of FAs decreased in concentration in meat with the inclusion of sainfoin. These results might not be desirable as some of the positive effects related to human health are attributed to BCFA intake.^51^

The higher concentrations of C18:3 n-3 obtained in the rumen of 40SF lambs produced a higher transference of this FA to animal tissues as previously reviewed with high PUFA n-3 diets.^7^ This is because higher concentrations of this FA would escape from the rumen, be absorbed in the small intestine, and be deposited in the muscle.^52^ Meat CLA c9,t11 concentrations of 40SF lambs were almost 40% higher than those in the rest of the diets, and enhancement of this FA is one of the most desirable goals in ruminant products.^53^ The CLA c9,t11 present in meat is derived from the following: (i) ruminal synthesis as a BH product of C18:2 n-6 and subsequent transport to meat or (ii) endogenous synthesis in the tissues from rumen-derived C18:1 t11 via Δ^9^-desaturase activity.^54^ As higher concentrations of C18:1 t11 were found in the 40SF rumen, it can be assumed that the increase in the percentage of CLA c9,t11 in the meat came predominantly from the higher ruminal concentration of C18:1 t11. In this case, the promotion of t11 BH pathways of both C18:3 n-3 and C18:2 n-6 and the greater BH extent of C18:3 n-3 allowed higher concentrations of C18:1 t11 in rumen, thus promoting its conversion to CLA c9,t11 in meat. This result agrees with previous studies stating that approximately 93% of C18:1 t11 is converted to CLA c9,t11 in lamb meat.^33^ A higher CLA c9,t11 concentration in lamb meat of forage-fed animals compared with that from a grain-based diet has been previously reported^14,55^ and showed an increase in CLA isomer formation due to a higher PUFA BH extent. However, despite the increase in CLA c9,t11 concentration with the presence of 40% sainfoin, the percentages of this FA with respect to total FA were low relative to those reported in other studies conducted with light lambs.^47,48^ This is probably because *trans*-C18, including CLA isomers, are preferentially incorporated into neutral lipids.^56^ Lean meat with a low FA content is mostly composed of membrane phospholipids (polar lipids); thus, both CLA c9,t11 and C18:1 t11 might not have been preferentially deposited in the IMF. Possibly for the same reason, no effect on C18:1 t11 was observed in the meat, even though differences were observed in the rumen. Therefore, this study shows that CLA c9,t11 promotion in meat should not only be focused on a high substrate supply coming from ruminal BH but should also be accompanied by a high IMF, rich in total FAs as stated in the work of Bessa et al.^54^

The high C20:5 n-3 and C22:6 n-3 concentrations found in 40SF muscle is an indication that, even with a higher BH of C18:3 n-3 occurring in the rumen of these animals, greater amounts of C18:3 n-3 were absorbed and made available for the synthesis of both C20:5 n-3 and C22:6 n-3 as C18:3 n-3 is the precursor for the synthesis of long-chain PUFAs n-3.^57^ Previous studies described similar results in muscle from lambs fed sainfoin silage^58^ and lambs grazed with forage legumes.^59^ Furthermore, the lowest PUFA n-6/PUFA n-3 values obtained in the 40SF group demonstrate that it is possible to decrease that value to within the recommended limits^60^ by including sainfoin in a concentrate-based diet.

In conclusion, the inclusion of sainfoin in the diet supported an increase in ruminal PUFA n-3 percentages and a decrease in PUFA n-6/PUFA n-3 and C18:1 t10/C18:1 t11 ratios, with this effect being most apparent in the 40SF diet. This diet also produced a major decrease in the percentage of PUFA n-6 and an increase in that of C18:1 t11 and CLA c9,t11 in the rumen. These ruminal changes were reflected in greater percentages of PUFA n-3 and CLA c9,t11 in the meat as the level of sainfoin inclusion increased, which resulted in higher amounts of those FAs in 40SF meat, even with a lower amount of total FA. Therefore, meat from lambs fed with 20 or 40% sainfoin in the diet achieved a healthier FA profile for humans. The more desirable results obtained in the 40SF diet could be related to a higher C18:3 n-3 intake due to the 40% inclusion of sainfoin in the diet along with a stronger t11 biohydrogenation pathway in the rumen of these lambs.

Therefore, the inclusion of sainfoin in lamb finishing concentrate improved meat quality, especially when sainfoin was included up to 40% as the effects were greater. Although the total amounts of beneficial FA achieved in meat from the inclusion of sainfoin in the concentrate were not as high as in several studies, where lambs were fed fresh, silage, or hay forages, this study demonstrates that it is possible to improve the FA profile of meat without modifying the typical southern European lamb production system, where lambs are concentrate-fattened indoors.

## Supporting Information Available

Results of total FA intake and individual fatty acid intake (Supplementary Table S1) (PDF)

## References

[ref1] Willett W.; Rockström J.; Loken B.; Springmann M.; Lang T.; Vermeulen S.; Garnett T.; Tilman D.; DeClerck F.; Wood A.; Jonell M.; Clark M.; Gordon L. J.; Fanzo J.; Hawkes C.; Zurayk R.; Rivera J. A.; De Vries W.; Majele Sibanda L.; Afshin A.; Chaudhary A.; Herrero M.; Agustina R.; Branca F.; Lartey A.; Fan S.; Crona B.; Fox E.; Bignet V.; Troell M.; Lindahl T.; Singh S.; Cornell S. E.; Srinath Reddy K.; Narain S.; Nishtar S.; Murray C. J. L. Food in the Anthropocene: The EAT–Lancet Commission on Healthy Diets from Sustainable Food Systems. Lancet 2019, 393, 447–492. 10.1016/S0140-6736(18)31788-4. 30660336

[ref2] Woods V. B.; Fearon A. M. Dietary Sources of Unsaturated Fatty Acids for Animals and Their Transfer into Meat, Milk and Eggs: A Review. Livest. Sci. 2009, 126, 1–20. 10.1016/j.livsci.2009.07.002.

[ref3] Vasta V.; Bessa R. J. B. Manipulating Ruminal Biohydrogenation by the Use of Plants Bioactive Compounds. In Dietary Phytochemicals and Microbes; Patra AK. K.., Ed. Springer: Dordrecht, The Netherlands, 2012; pp 263–284.

[ref4] Jenkins T. C.; Wallace R. J.; Moate P. J.; Mosley E. E. Board-Invited Review: Recent Advances in Biohydrogenation of Unsaturated Fatty Acids within the Rumen Microbial Ecosystem. J. Anim. Sci. 2008, 86, 397–412. 10.2527/jas.2007-0588. 18042812

[ref5] Parodi P. W. Dietary Guidelines for Saturated Fatty Acids Are Not Supported by the Evidence. Int. Dairy J. 2016, 52, 115–123. 10.1016/j.idairyj.2015.08.007.

[ref6] Vahmani P.; Ponnampalam E. N.; Kraft J.; Mapiye C.; Bermingham E. N.; Watkins P. J.; Proctor S. D.; Dugan M. E. R. Bioactivity and Health Effects of Ruminant Meat Lipids. Invited Review. Meat Sci. 2020, 165, 108114 10.1016/j.meatsci.2020.108114. 32272342

[ref7] Álvarez-Rodríguez J.; Urrutia O.; Lobón S.; Ripoll G.; Bertolín J. R.; Joy M. Insights into the Role of Major Bioactive Dietary Nutrients in Lamb Meat Quality: A Review. J. Animal Sci. Biotechnol. 2022, 13, 1–16. 10.1186/s40104-021-00665-0. PMC8819927 35125115

[ref8] Shingfield K. J.; Bonnet M.; Scollan N. D. Recent Developments in Altering the Fatty Acid Composition of Ruminant-Derived Foods. Animal. 2013, 7, 132–162. 10.1017/S1751731112001681. 23031638

[ref9] Moorby J. M.; Fraser M. D. New Feeds and New Feeding Systems in Intensive and Semi-Intensive Forage-Fed Ruminant Livestock Systems. Animal. 2021, 100297 10.1016/j.animal.2021.100297. 34312094 PMC8664714

[ref10] Santos-Silva J.; Alves S. P.; Francisco A.; Portugal A. P.; Dentinho M. T.; Almeida J.; da Silva J. L. R.; Fialho L.; Cachucho L.; Jerónimo E.; Barradas A.; Rodrigues A.; Rodrigues N.; Teixeira R. F. M.; Domingos T.; Bessa R. J. B. Forage Based Diet as an Alternative to a High Concentrate Diet for Finishing Young Bulls - Effects on Growth Performance, Greenhouse Gas Emissions and Meat Quality. Meat Sci. 2023, 198, 109098 10.1016/j.meatsci.2023.109098. 36681060

[ref11] Griinari J. M.; Dwyer D. A.; Mcguire M. A.; Bauman D. E.; Palmquist D. L.; Nurmela K. V. V. Trans-Octadecenoic Acids and Milk Fat Depression in Lactating Dairy Cows. J. Dairy Sci. 1998, 81, 1251–1261. 10.3168/jds.S0022-0302(98)75686-3. 9621226

[ref12] Rufino-Moya P. J.; Bertolín J. R.; Blanco M.; Lobón S.; Joy M. Fatty Acid Profile, Secondary Compounds and Antioxidant Activities in the Fresh Forage, Hay and Silage of Sainfoin (Onobrychis Viciifolia) and Sulla (Hedysarum Coronarium). J. Sci. Food Agric. 2022, 102, 4736–4743. 10.1002/jsfa.11834. 35195298 PMC9542018

[ref13] Frutos P.; Hervás G.; Natalello A.; Luciano G.; Fondevila M.; Priolo A.; Toral P. G. Ability of Tannins to Modulate Ruminal Lipid Metabolism and Milk and Meat Fatty Acid Profiles. Anim. Feed Sci. Technol. 2020, 269, 114623 10.1016/j.anifeedsci.2020.114623.

[ref14] Lobón S.; Blanco M.; Sanz A.; Ripoll G.; Bertolín J. R.; Joy M. Meat Quality of Light Lambs Is More Affected by the Dam’s Feeding System during Lactation than by the Inclusion of Quebracho in the Fattening Concentrate. J. Anim. Sci. 2017, 95, 4998–5011. 10.2527/jas2017.1595. 29293726 PMC6292321

[ref15] Baila C.; Joy M.; Blanco M.; Casasús I.; Ripoll G.; Lobón S. Sainfoin Can Be Included up to 40% in the Fattening Concentrate of Lambs without Impairing Their Performance, Ruminal Fermentation and Carcass Quality. Under Review 2023, 0.

[ref16] Baila C.; Joy M.; Blanco M.; Casasús I.; Bertolín J. R.; Lobón S. Effects of Feeding Sainfoin Proanthocyanidins to Lactating Ewes on Intake, Milk Production and Plasma Metabolites. Animal 2022, 100438 10.1016/j.animal.2021.100438. 34996024

[ref17] Bertolín J. R.; Joy M.; Blanco M. Malondialdehyde Determination in Raw and Processed Meat Products by UPLC-DAD and UPLC-FLD. Food Chem. 2019, 298, 125009 10.1016/j.foodchem.2019.125009. 31260970

[ref18] Lee M. R. F.; Tweed J. K. S.; Kim E. J.; Scollan N. D. Beef, chicken and lamb fatty acid analysis—a simplified direct bimethylation procedure using freeze-dried material. Meat Sci. 2012, 92, 863–866. 10.1016/j.meatsci.2012.06.013. 22749429

[ref19] Kramer J. K. G.; Fellner V.; Dugan M. E. R.; Sauer F. D.; Mossoba M. M.; Yurawecz M. P. Evaluating Acid and Base Catalysts in the Methylation of Milk and Rumen Fatty Acids with Special Emphasis on Conjugated Dienes and Total Trans Fatty Acids. Lipids 1997, 32, 1219–1228. 10.1007/s11745-997-0156-3. 9397408

[ref20] Alves S. P.; Bessa R. J. B. Comparison of Two Gas–Liquid Chromatograph Columns for the Analysis of Fatty Acids in Ruminant Meat. J. Chromatogr. A 2009, 1216, 5130–5139. 10.1016/j.chroma.2009.04.079. 19446820

[ref21] Bravo-Lamas L.; Barron L. J. R.; Kramer J. K. G.; Etaio I.; Aldai N. Characterization of the Fatty Acid Composition of Lamb Commercially Available in Northern Spain: Emphasis on the Trans-18:1 and CLA Content and Profile. Meat Sci. 2016, 117, 108–116. 10.1016/j.meatsci.2016.02.043. 26970291

[ref22] Alves S. P.; Francisco A.; Costa M.; Santos-Silva J.; Bessa R. J. B. Biohydrogenation Patterns in Digestive Contents and Plasma of Lambs Fed Increasing Levels of a Tanniferous Bush (Cistus Ladanifer L.) and Vegetable Oils. Anim. Feed Sci. Technol. 2017, 225, 157–172. 10.1016/j.anifeedsci.2017.01.018.

[ref23] Alves S. P.; Santos-Silva J.; Cabrita A. R. J.; Fonseca A. J. M.; Bessa R. J. B. Detailed Dimethylacetal and Fatty Acid Composition of Rumen Content from Lambs Fed Lucerne or Concentrate Supplemented with Soybean Oil. PLoS One 2013, 8, e58386 10.1371/journal.pone.0058386. 23484024 PMC3587585

[ref24] Alves S. P.; Mendonça S. H.; Silva J. L.; Bessa R. J. B. Nannochloropsis Oceanica, a Novel Natural Source of Rumen-Protected Eicosapentaenoic Acid (EPA) for Ruminants. Sci. Rep. 2018, 8, 1–10. 10.1038/s41598-018-28576-7. 29980726 PMC6035222

[ref25] Alves S. P.; Bessa R. J. B. The Trans-10,Cis-15 18:2: A Missing Intermediate of Trans-10 Shifted Rumen Biohydrogenation Pathway?. Lipids 2014, 49, 527–541. 10.1007/s11745-014-3897-4. 24677182

[ref26] Baila C.; Joy M.; Bertolín J. R.; Blanco M.; Casasús I.; Lobón S. Effect of Sainfoin Proanthocyanidins on Milk Fatty Acids from Ewes Rearing Suckling Lambs. Animal 2023, 17, 100862 10.1016/j.animal.2023.100862. 37285648

[ref27] Fievez V.; Colman E.; Castro-Montoya J. M.; Stefanov I.; Vlaeminck B. Milk Odd-and Branched-Chain Fatty Acids as Biomarkers of Rumen Function—An Update. Anim. Feed Sci. Technol. 2012, 172, 51–65. 10.1016/j.anifeedsci.2011.12.008.

[ref28] Costa M.; Alves S. P.; Cabo Â.; Guerreiro O.; Stilwell G.; Dentinho M. T.; Bessa R. J. B. Modulation of in Vitro Rumen Biohydrogenation by Cistus Ladanifer Tannins Compared with Other Tannin Sources. J. Sci. Food Agric. 2017, 97, 629–635. 10.1002/jsfa.7777. 27130817

[ref29] Kishino S.; Takeuchi M.; Park S. B.; Hirata A.; Kitamura N.; Kunisawa J.; Kiyono H.; Iwamoto R.; Isobe Y.; Arita M.; Arai H.; Ueda K.; Shima J.; Takahashi S.; Yokozeki K.; Shimizu S.; Ogawa J. Polyunsaturated Fatty Acid Saturation by Gut Lactic Acid Bacteria Affecting Host Lipid Composition. Proc. Natl. Acad. Sci. U.S.A. 2013, 110, 17808–17813. 10.1073/pnas.1312937110. 24127592 PMC3816446

[ref30] Toral P. G.; Hervás G.; Missaoui H.; Andrés S.; Giráldez F. J.; Jellali S.; Frutos P. Effects of a Tannin-Rich Legume (Onobrychis Viciifolia) on in Vitro Ruminal Biohydrogenation and Fermentation. Span. J. Agric. Res. 2016, 14, e0602 10.5424/sjar/2016141-8989.

[ref31] Huyen N. T.; Verstegen M. W. A.; Hendriks W. H.; Pellikaan W. F. Sainfoin (Onobrychis Viciifolia) Silage in Dairy Cow Rations Reduces Ruminal Biohydrogenation and Increases Transfer Efficiencies of Unsaturated Fatty Acids from Feed to Milk. Anim. Nutr. 2020, 6, 333–341. 10.1016/j.aninu.2020.05.001. 33005767 PMC7503786

[ref32] Alves S. P.; Vahmani P.; Mapiye C.; McAllister T. A.; Bessa R. J.; Dugan M. E. Trans-10 18:1 in Ruminant Meats: A Review. Lipids 2021, 56, 539–562. 10.1002/lipd.12324. 34608647

[ref33] Palmquist D. L.; St-Pierre N.; Mcclure K. E. Tissue Fatty Acid Profiles Can Be Used to Quantify Endogenous Rumenic Acid Synthesis in Lambs. J. Nutr. 2004, 134, 2407–2414. 10.1093/jn/134.9.2407. 15333736

[ref34] Oliveira M. A.; Alves S. P.; Santos-Silva J.; Bessa R. J. Effects of clays used as oil adsorbents in lamb diets on fatty acid composition of abomasal digesta and meat. Anim. Feed Sci. Technol. 2016, 213, 64–73. 10.1016/j.anifeedsci.2016.01.006.

[ref35] Griinari J. M.; Bauman D. E. Biosynthesis of Conjugated Linoleic Acid and Its Incorporation into Meat and Milk in Ruminants. In Advances in conjugated linoleic acid research; Yurawecz M., Mossoba M., Kramer J., Pariza M., Nelson G., Eds.; AOCS Press: Champaign, IL, 1999; 1, pp180–200.

[ref36] Tricon S.; Burdge G. C.; Kew S.; Banerjee T.; Russell J. J.; Jones E. L.; Grimble R. F.; Williams C. M.; Yaqoob P.; Calder P. C. Opposing Effects of Cis-9,Trans-11 and Trans-10,Cis-12 Conjugated Linoleic Acid on Blood Lipids in Healthy Humans. Am. J. Clin. Nutr. 2004, 80, 614–620. 10.1093/ajcn/80.3.614. 15321800

[ref37] Kucuk O.; Hess B. W.; Ludden P. A.; Rule D. C. Effect of forage: concentrate ratio on ruminal digestion and duodenal flow of fatty acids in ewes. J. Anim. Sci. 2001, 79, 2233–2240. 10.2527/2001.7982233x. 11518234

[ref38] Bessa R. J. B.; Portugal P. V.; Mendes I. A.; Santos-Silva J. Effect of lipid supplementation on growth performance, carcass and meat quality and fatty acid composition of intramuscular lipids of lambs fed dehydrated lucerne or concentrate. Livest. Prod. Sci. 2005, 96, 185–194. 10.1016/j.livprodsci.2005.01.017.

[ref39] Jenkins T. C. Advances in Ruminant Lipid Metabolism. J. Dairy Sci. 1993, 76, 3851–3863. 10.3168/jds.S0022-0302(93)77727-9. 8132891

[ref40] Maia M. R. G.; Chaudhary L. C.; Figueres L.; Wallace R. J. Metabolism of Polyunsaturated Fatty Acids and Their Toxicity to the Microflora of the Rumen. Anton. Leeuw. Int. J. 2007, 91, 303–314. 10.1007/s10482-006-9118-2. 17072533

[ref41] Sackmann J. R.; Duckett S. K.; Gillis M. H.; Realini C. E.; Parks A. H.; Eggelston R. B. Effects of Forage and Sunflower Oil Levels on Ruminal Biohydrogenation of Fatty Acids and Conjugated Linoleic Acid Formation in Beef Steers Fed Finishing Diets. J. Anim. Sci. 2003, 81, 3174–3181. 10.2527/2003.81123174x. 14677873

[ref42] Grosse Brinkhaus A. G.; Bee G.; Silacci P.; Kreuzer M.; Dohme-Meier F. Effect of Exchanging Onobrychis Viciifolia and Lotus Corniculatus for Medicago Sativa on Ruminal Fermentation and Nitrogen Turnover in Dairy Cows. J. Dairy Sci. 2016, 99, 4384–4397. 10.3168/jds.2015-9911. 26995129

[ref43] Campidonico L.; Toral P. G.; Priolo A.; Luciano G.; Valenti B.; Hervás G.; Frutos P.; Copani G.; Ginane C.; Niderkorn V. Fatty Acid Composition of Ruminal Digesta and Longissimus Muscle from Lambs Fed Silage Mixtures Including Red Clover, Sainfoin, and Timothy. J. Anim. Sci. 2016, 94, 1550–1560. 10.2527/jas.2015-9922. 27136014

[ref44] Bauchart D. Lipid Absorption and Transport in Ruminants. J. Dairy Sci. 1993, 76, 3864–3881. 10.3168/jds.S0022-0302(93)77728-0. 8132892

[ref45] Vasta V.; Mele M.; Serra A.; Scerra M.; Luciano G.; Lanza M.; Priolo A. Metabolic Fate of Fatty Acids Involved in Ruminal Biohydrogenation in Sheep Fed Concentrate or Herbage with or without Tannins. J. Anim. Sci. 2009, 87, 2674–2684. 10.2527/jas.2008-1761. 19395521

[ref46] Christie W. W. The Effects of Diet and Other Factors on the Lipid Composition of Ruminant Tissues and Milk. In Lipid Metabolism in Ruminant Animals; Christie W. W. Ed.; Elsevier, 1981; pp 193–226.

[ref47] Natalello A.; Luciano G.; Morbidini L.; Valenti B.; Pauselli M.; Frutos P.; Biondi L.; Rufino-Moya P. J.; Lanza M.; Priolo A. Effect of Feeding Pomegranate Byproduct on Fatty Acid Composition of Ruminal Digesta, Liver, and Muscle in Lambs. J. Agric. Food Chem. 2019, 67, 4472–4482. 10.1021/acs.jafc.9b00307. 30929432

[ref48] Blanco M.; Ripoll G.; Lobón S.; Bertolín J. R.; Casasús I.; Joy M. The Inclusion of Pea in Concentrates Had Minor Effects on the Meat Quality of Light Lambs. Animals 2021, 11, 2385 10.3390/ani11082385. 34438842 PMC8388810

[ref49] Vasta V.; Priolo A.; Scerra M.; Hallett K. G.; Wood J. D.; Doran O. Δ9 Desaturase Protein Expression and Fatty Acid Composition of Longissimus Dorsi Muscle in Lambs Fed Green Herbage or Concentrate with or without Added Tannins. Meat Sci. 2009, 82, 357–364. 10.1016/j.meatsci.2009.02.007. 20416712

[ref50] Jerónimo E.; Alves S. P.; Dentinho M. T. P.; Martins S. V.; Prates J. A. M.; Vasta V.; Santos-Silva J.; Bessa R. J. B. Effect of Grape Seed Extract, Cistus Ladanifer L., and Vegetable Oil Supplementation on Fatty Acid Composition of Abomasal Digesta and Intramuscular Fat of Lambs. J. Agric. Food Chem. 2010, 58, 10710–10721. 10.1021/jf1021626. 20831248

[ref51] Ran-Ressler R. R.; Khailova L.; Arganbright K. M.; Adkins-Rieck C. K.; Jouni Z. E.; Koren O.; Ley R. E.; Brenna J. T.; Dvorak B. Branched Chain Fatty Acids Reduce the Incidence of Necrotizing Enterocolitis and Alter Gastrointestinal Microbial Ecology in a Neonatal Rat Model. PLoS One 2011, 6, e29032 10.1371/journal.pone.0029032. 22194981 PMC3237582

[ref52] Scollan N.; Hocquette J. F.; Nuernberg K.; Dannenberger D.; Richardson I.; Moloney A. Innovations in Beef Production Systems That Enhance the Nutritional and Health Value of Beef Lipids and Their Relationship with Meat Quality. Meat Sci. 2006, 74, 17–33. 10.1016/j.meatsci.2006.05.002. 22062713

[ref53] Palmquist D. L. Milk Fat: Origin of Fatty Acids and Influence of Nutritional Factors Thereon. In Advanced dairy chemistry; Springer, 2006; 2, pp 43–92.

[ref54] Bessa R. J. B.; Alves S. P.; Santos-Silva J. Constraints and Potentials for the Nutritional Modulation of the Fatty Acid Composition of Ruminant Meat. Eur. J. Lipid Sci. Technol. 2015, 117, 1325–1344. 10.1002/ejlt.201400468.

[ref55] Álvarez-Rodríguez J.; Ripoll G.; Lobón S.; Sanz A.; Blanco M.; Joy M. Alfalfa but Not Milk in Lamb’s Diet Improves Meat Fatty Acid Profile and α-Tocopherol Content. Food Res. Int. 2018, 107, 708–716. 10.1016/j.foodres.2018.03.007. 29580538

[ref56] Jerónimo E.; Alves S. P.; Alfaia C. M.; Prates J. A. M.; Santos-Silva J.; Bessa R. J. B. Biohydrogenation Intermediates Are Differentially Deposited between Polar and Neutral Intramuscular Lipids of Lambs. Eur. J. Lipid Sci. Technol. 2011, 113, 924–934. 10.1002/ejlt.201000398.

[ref57] Scollan N. D.; Dannenberger D.; Nuernberg K.; Richardson I.; MacKintosh S.; Hocquette J. F.; Moloney A. P. Enhancing the Nutritional and Health Value of Beef Lipids and Their Relationship with Meat Quality. Meat Sci. 2014, 97, 384–394. 10.1016/j.meatsci.2014.02.015. 24697921

[ref58] Girard M.; Dohme-Meier F.; Silacci P.; Ampuero Kragten S.; Kreuzer M.; Bee G. Forage Legumes Rich in Condensed Tannins May Increase N-3 Fatty Acid Levels and Sensory Quality of Lamb Meat. J. Sci. Food Agric. 2016, 96, 1923–1933. 10.1002/jsfa.7298. 26059039

[ref59] Gruffat D.; Durand D.; Rivaroli D.; Do Prado I. N.; Prache S. Comparison of Muscle Fatty Acid Composition and Lipid Stability in Lambs Stall-Fed or Pasture-Fed Alfalfa with or without Sainfoin Pellet Supplementation. Animal 2020, 14, 1093–1101. 10.1017/S1751731119002507. 31658927

[ref60] Vahmani P.; Mapiye C.; Prieto N.; Rolland D. C.; McAllister T. A.; Aalhus J. L.; Dugan M. E. R. The Scope for Manipulating the Polyunsaturated Fatty Acid Content of Beef: A Review. J. Animal Sci. Biotechnol. 2015, 6, 1–13. 10.1186/s40104-015-0026-z. PMC4509462 26199725

