# Supplementary material for: Inclusion of Sainfoin in the Concentrate of Finishing Lambs: Fatty Acid Profiles of Rumen, Plasma, and Muscle
Source: J Agric Food Chem. 2023 Nov 13;71(46):17947–58. doi: 10.1021/acs.jafc.3c05902 (PMC10682985; doi:10.1021/acs.jafc.3c05902)
Supplement: Supplementary file 1 — jf3c05902_si_001.pdf [file jf3c05902_si_001.pdf]

SUPPORITNG INFORMATION

Inclusion of sainfoin in the concentrate of finishing lambs: Fatty acid profile of rumen, plasma, and muscle

Clàudia Baila [1,2], Margalida Joy [1,2] *, Juan Ramón Bertolín [1,2], Susana Alves [3,4], Rui Bessa [3,4], Mireia Blanco [1,2], and Sandra Lobón [1,2]

[1] Departamento de Ciência Animal. Centro de Investigación y Tecnología Agroalimentaria de Aragón (CITA). Avda. Montañana 930, 50059, Zaragoza, España

[2] Instituto Agroalimentario de Aragón – IA2 (CITA-Universidad de Zaragoza). Zaragoza, España

[3] CIISA, Centro de Investigação Interdisciplinar em Sanidade Animal, Faculdade de Medicina Veterinária, Universidade de Lisboa, 1300-477 Lisboa, Portugal

[4] Laboratório Associado para Ciência Animal e Veterinária (AL4AnimalS), Avenida da Universidade Técnica, 1300-477 Lisboa, Portugal

*Corresponding author:

Margalida Joy

Centro de Investigación y Tecnología Agroalimentaria de Aragón (CITA). Avda. Montañana 930, 50059, Zaragoza, España.

Tel: +34 976716442

*Email: mjoyt@unizar.es

Supplementary Table S1. Effect of the inclusion of sainfoin in the finishing concentrate on the intake of fatty acids (FA) of the concentrates.

	diets [a]		
	0SF	20SF	40SF
total fatty acid (FA) intake, g/d	33.0	34.2	41.4
Intake individual FA, g/d			
C12:0	0.03	0.02	0.05
C14:0	0.17	0.17	0.25
C16:0	9.12	9.57	12.43
C16:1 c9	0.07	0.08	0.11
C18:0	2.43	2.39	3.07
C18:1 c9	8.13	8.24	11.11
C18:1 c11	0.09	0.09	0.12
C18:2 n-6	12.16	11.97	11.52
C18:3 n-3	0.82	1.63	2.78

[a] 0SF, 0% of sainfoin; 20SF, 20% of sainfoin; 40SF, 40% of sainfoin in the finishing concentrate.
